# High Q-factor reconfigurable microresonators induced in side-coupled optical fibres

**DOI:** 10.1038/s41377-023-01247-7

**Published:** 2023-08-18

**Authors:** Victor Vassiliev, Michael Sumetsky

**Affiliations:** Aston Institute of Photonic Technologies, Aston University, Birmingham, B4 7ET UK

**Keywords:** Micro-optics, Optical metrology

## Abstract

High Q-factor monolithic optical microresonators found numerous applications in classical and quantum optical signal processing, microwave photonics, ultraprecise sensing, as well as fundamental optical and physical sciences. However, due to the solid structure of these microresonators, attaining the free spectral range tunability of most of them, critical for several of these applications, was, so far, unfeasible. To address this problem, here we experimentally demonstrate that the side-coupling of coplanar bent optical fibres can induce a high Q-factor whispering gallery mode optical microresonator. By changing the curvature radius of fibres from the centimetre order to the millimetre order, we demonstrate fully mechanically reconfigurable optical microresonators with dimensions varying from the millimetre order to 100-micron order and free spectral range varying from a picometre to ten picometre order. The developed theory describes the formation of the discovered microresonators and their major properties in a reasonable agreement with the experimental data. The new microresonators may find applications in cavity QED, microresonator optomechanics, frequency comb generation with tuneable repetition rate, tuneable lasing, and tuneable processing and delay of optical pulses.

## Introduction

Microphotonic devices and circuits commonly consist of one or multiple connected basic elements, such as waveguides, couplers, and microresonators^1,2^. In addition to the requirements of high fabrication precision and low losses^2,3^, the tunability of these circuits and devices is of critical importance for a variety of applications^4,5^. While more complex tuneable microphotonics circuits may be designed to enable wide class of programmable transformations of optical signals (see e.g. in ref. ^1^), simple microdevices, such as standing along tuneable three-dimensional microresonators, allow for unique functionalities not possible to achieve by other means. For a variety of applications, the tunability of spherical, toroidal, and bottle microresonators has been demonstrated using mechanical stretching, heating, and nonlinear light effects including those in monolithic and specially coated microresonators^6–10^. In most of these approaches, it is only possible to tune series of wavelength eigenvalues simultaneously without noticeable change in their separation.

However, for several applications, which include cavity QED^8,11,12^, optomechanics^13,14^, frequency microcomb generation^15,16^, optical signal processing and delay^4,5,17^, and lasing^18–21^, it is critical to have microresonators with tuneable eigenwavelength separation and, in particular, with tuneable free spectral range. For example, the latter allows the creation of optical frequency microcomb generators and microlasers with continuously tuneable repetition rate and wavelength and to tune the microresonator eigenfrequency separation in resonance with the frequency of its mechanical oscillations. Considerable variation of the eigenwavelength separation commonly requires the variation of microresonator dimension or its refractive index parameters by the quantity comparable with their original values. One approach to solve this problem consists in using Fabry-Perot microresonators with tuneable mirror separation which contain the optical materials under interest^12,21,22^. The additional flexibility of tuning can be achieved by employing Fabry-Perot microresonators with a liquid material inside^21^ or translating a wedge-shaped solid optical material to vary its dimensions inside the Fabry-Perot microresonator^23^.

Alternatively, of special interest is attaining the eigenwavelength separation tunability in three-dimensional monolithic high Q-factor microresonators, e.g. those with spherical, toroidal, and bottle shapes. This will allow us to add tunability to the emerging applications of these microresonators in QED, optomechanics, lasing, and frequency comb generation noted above. However, the deformation of most of these monolithic microresonators to achieve significant change of their eigenwavelength separation is unfeasible.

A unique exception, though, is exhibited by SNAP (Surface Nanoscale Axial Photonics) microresonators^24^. These microresonators are introduced at the surface of an optical fibre by its nanoscale deformation, which causes the nanoscale variation of the cutoff wavelengths (CWLs) controlling the slow propagation of whispering gallery modes (WGMs) along the fibre axis (see^24,25^ and references therein). In Ref. ^26^, a SNAP microresonator induced and fully reconfigurable by local heating of an optical fibre was demonstrated. In Ref. ^27^, it was shown that it is possible to create a SNAP microresonator and control its dimensions by local bending of an optical fibre. Both approaches allow for tuning of eigenwavelength separation of microresonators by the quantity comparable to or larger than its original value. However, in both approaches, the induced microresonator shapes had limited flexibility, and their characteristic axial dimensions could not be reduced below several millimetres. In the first case, this restriction was caused by the imposed length of the characteristic heat distribution along the fibre. In the second case, the reduction of microresonator size was limited by the smallest curvature radius corresponding to the fibre breakage threshold.

**Fig. 1:**
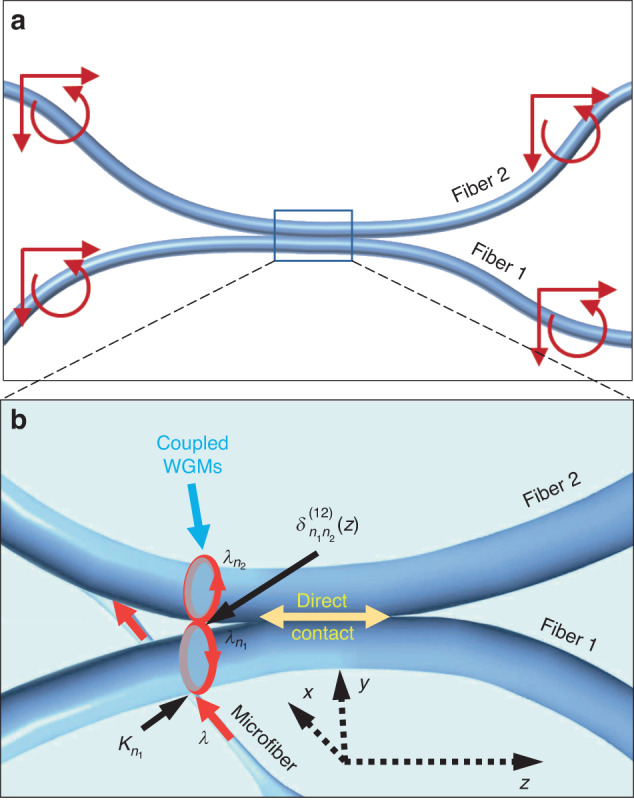
Side-coupled bent optical fibres. **a** Coplanar bent optical fibres touching each other. The fibre profile is manipulated by bending and translating the fibre tails indicated by curved and straight arrows. **b** Illustration of coupling between the input-output microfibre and WGMs in Fibre 1 and Fibre 2 near cutoff wavelengths

In this paper we report on our discovery of a new type of WGM optical microresonators which belongs to the group of SNAP microresonators. We show that side-coupled coplanar bent fibres (Fig. 1) can induce a high Q-factor SNAP microresonator localised in the region of fibre coupling. The configuration of fibres shown in Fig. 1 allows us to flexibly tune the shape of the induced SNAP microresonators and their axial dimensions from hundred micron order to millimetre order and, respectively, tune their eigenwavelength separation from ten picometre order to picometre order.

## Results

### Cutoff wavelengths of uncoupled and side-coupled straight fibres

**Fig. 2:**
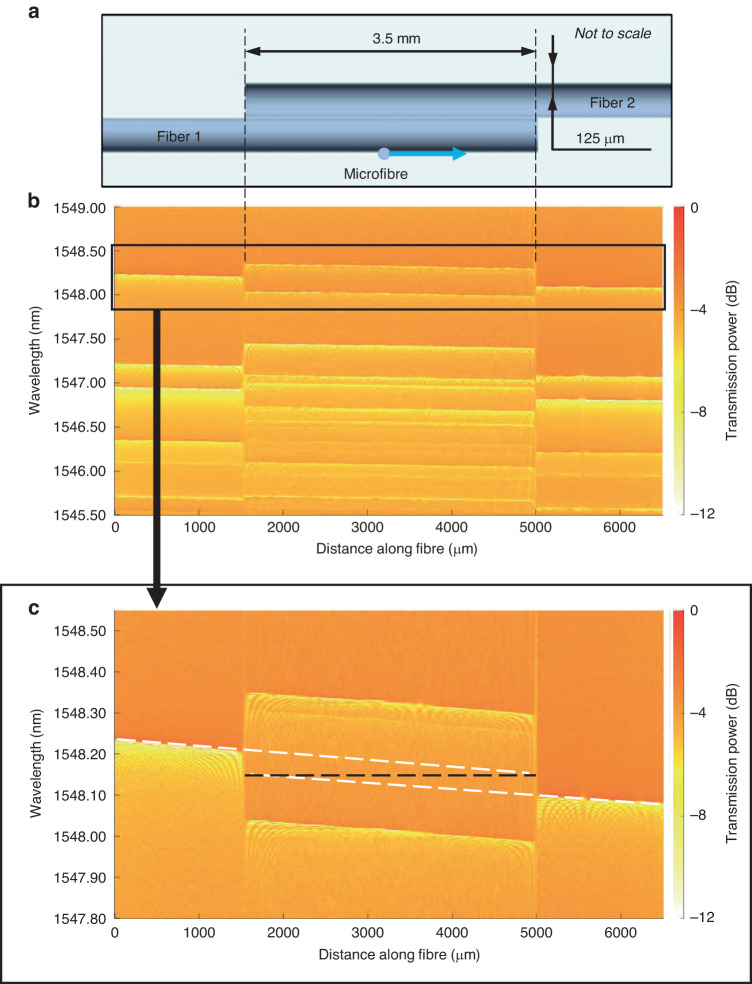
CWL splitting in side-coupled straight optical fibres. **a** Illustration of side-coupled straight optical fibre configuration. **b** Spectrogram of this configuration. Here and in all spectrograms below we calibrate the output power relative to its maximum value at the spectrogram. **c** Magnified section outlined in the spectrogram (**b**)

First, it is instructive to consider the behaviour of CWLs for uncoupled and side-coupled *straight* optical fibres. For this purpose, we cleave a 125-micron diameter uncoated commercial optical fibre into two pieces (Fibre 1 and Fibre 2), which are then coaxially aligned and put into contact along 3.5 mm of their length, as shown in Fig. 2a. Light is launched into Fibre 1 by a transversely oriented taper with the micrometre diameter waist (input-output microfibre) connected to the Optical Spectrum Analyzer (OSA). After coupling into Fibre 1, light forms WGMs propagating along the fibre surface. In the region of direct contact of fibres (Fig. 2a), WGMs in Fibre 1 and Fibre 2 are coupled to each other.

To characterise the effect of interfibre coupling, we measured the spectrograms of the configured fibre system. For this purpose, the input-output microfibre was translated along Fibre 1 (Figs. 1b and 2a), touching it periodically with the spatial resolution of 2 µm. At the cut end of Fibre 1, the microfibre was moved toward Fibre 2 and continued scanning Fibre 2. The spectrograms of transmission power *P*(*λ,z*) were measured as a function of wavelength *λ* and microfibre position *z* along the axis of Fibre 1.

The measured spectrogram of our fibre system is shown in Fig. 2b. The left- and right-hand sides of this spectrogram show the spectrograms of uncoupled Fibre 1 and Fibre 2, respectively. Lines in the spectrogram shown in Fig. 2b indicate the CWLs of uncoupled and coupled fibres. These CWLs correspond to WGMs with different azimuthal and radial quantum numbers. The magnified copy of the section outlined in Fig. 2b is shown in Fig. 2c. It is seen that the CWLs appear as straight lines slightly tilted with respect to the horizontal direction. From the measured magnitude of tilt, *ε*_*t*_ = 0.015 nm/mm, we determine the linear variation of the fibre radius ∆*r*_*t*_ = *r*_0_*ε*_*t*_/*λ*_0_ = 0.6 nm/mm^28^. In the latter rescaling relation, we used *r*_0_ = 62.5 µm and *λ*_0_ = 1.55 nm. By linear extrapolation of CWLs of Fibre 1 and Fibre 2 (dashed white lines), we confirm that, as expected, their positions (horizontal black dashed line) coincide at the cut ends of these fibres.

Along the 3.5 mm contact region, WGMs in Fibre 1 couple to WGMs in Fibre 2 and the corresponding CWLs split. The structure and positions of CWLs in the contact area depend on the coupling magnitude and will be further discussed below. Here we note that the value of CWL splitting found, e.g. from Fig. 2c is ~0.1 nm, which coincides with characteristic values of CWL variation in SNAP microresonators^24,25^. In particular, the positive CWL shift in the coupling region leads to the WGM localisation and creation of a microresonator which can be tuned by changing the length of the side-coupled fibre segment. In our current experiment, the Q-factor of the induced SNAP resonator was poor due to the scattering of light at the imperfectly cleaved fibre ends, which typically ensures around 70% WGM reflectivity^29^. Nevertheless, we suggest that the demonstrated resonator can be directly used to create miniature broadly tuneable optical delay lines generalising our previous results based on the SNAP microresonators with fixed dimensions^30,31^. Indeed, in these devices the WGM pulses complete only a single round trip along the fibre axis. Therefore, the light attenuation at the fibre facets may reduce the output light power by around 50% only. We also suggest that, after feasible improvement, the Q-factor of these microresonators can be significantly enhanced as further discussed below.

### Basic experiment

In our proof-of-concept experiments, we used 125-micron diameter uncoated commercial silica optical fibres touching each other as shown in Fig. 1a. The ends of Fibre 1 and Fibre 2 were bent and translated to arrive at the required profile of these fibres near their coupling region illustrated in Fig. 1b. The fibres used were either originally straight or preliminary softened in a flame and bent permanently. As described in the previous section, WGMs were launched into Fibre 1 by a transversely oriented microfibre connected to the OSA. If the separation between Fibre 1 and Fibre 2 is small enough, WGMs penetrate from Fibre 1 into Fibre 2.

**Fig. 3:**
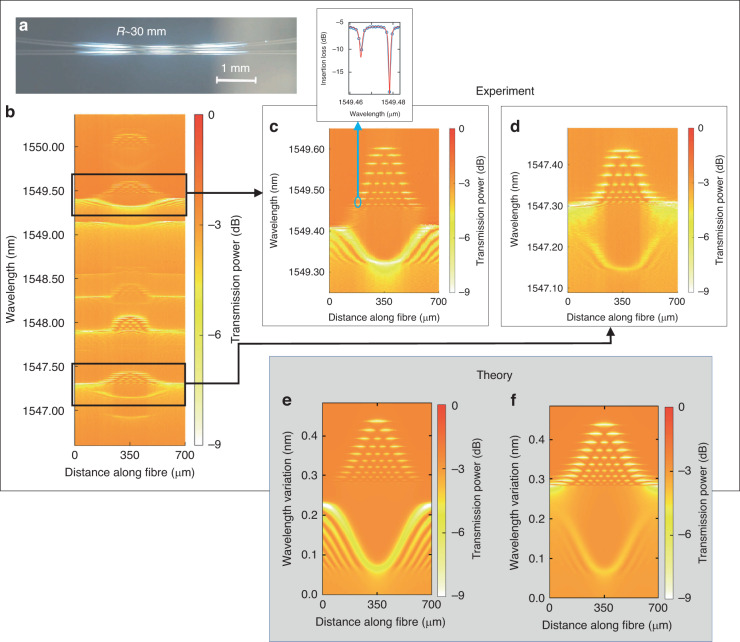
MIcroresonators created at side-coupled bent and straight optical fibres: experiment vs. theory. **a** Photograph of the side-coupled fibres used in the experiment. The upper fibre is bent with a curvature radius *R* ~ 30 mm and the lower fibre has a curvature radius greater than 1 m. **b** The spectrogram measured in the vicinity of the coupling region of these fibres. **c**, **d** Spectrograms showing the magnified sections outlined in spectrogram (**b**). **e**, **f** Spectrograms of the microresonators numerically found in the two-mode approximation detailed in the text, which replicate the experimental spectrograms in Fig. (**c** and **d**), respectively

In the simplest configuration considered in this Section, Fibre 1 was straight, and coplanar Fibre 2 was bent. The fibres were put in contact and then slightly pushed toward each other to increase the coupling region. The photograph of the fibre configuration used in this experiment is shown in Fig. 3a. From this picture, we estimated the curvature radius of the bent fibre as *R* ~ 30 mm (see further discussion of the fibre profile below). Figure. 3b shows the spectrogram of the configured structure measured along the 3.5 nm bandwidth within the 700 µm axial length of Fibre 1. At the edges of the scanned region, the interfibre coupling is negligible. In these regions, CWLs do not noticeably change with distance *z* and, thus, correspond to Fibre 1 only. The arrangement of CWLs in these regions is similar to that in Fig. 2b.

The effect of coupling shows up in the central region of the spectrogram in Fig. 3b. In this region, different CWLs exhibit different positive and negative variations along the axial length *z*. The exemplary regions of this spectrogram are magnified in Fig. 3c and d. It is seen that, as expected, in contrast to negative variations, positive CWL variations lead to WGM confinement and the creation of microresonators. Our estimates illustrated in the inset of Fig. 3c show that the Q-factor of the created microresonator (which measurement was limited by the 1.3 pm resolution of the OSA used) exceeds 10^6^. The observed CWL variations in Fig. 3c and d can be explained by the theory described below.

### Basic theory

We assume that the fibre bending is small enough so that the propagation of light along the axial direction of side-coupled fibres (Fig. 1b) can be considered as propagation along a single waveguide with an asymmetric cross-section including both fibres. The wavelengths of slow WGMs are close to the CWLs *λ*_*n*_(*z*) of this compound waveguide. To determine the complex-valued CWLs *λ*_*n*_(*z*), we introduce the original CWLs ${\lambda }_{1{n}_{1}}+\frac{i}{2}{\gamma }_{1{n}_{1}}$ and ${\lambda }_{2{n}_{2}}+\frac{i}{2}{\gamma }_{2{n}_{2}}$ of unbent Fibre 1 and Fibre 2 with the imaginary parts determined primarily by material losses and scattering of light at the fibre surface. We assume that there are *N*_1_ and *N*_2_ CWLs in Fibres 1 and Fibre 2, respectively, which contribute to the resonant transmission, so that *n*_*j*_ = 1,2,…, *N*_*j*_, *j* = 1,2. We refer to the integers *n*, *n*_1_ and *n*_2_ as to the transverse quantum numbers. Variation of *λ*_*n*_(*z*) is caused by the bending of fibres^27^ and, in our case, primarily by their coupling. In the absence of the input-output fibre, the CWLs of our system, *λ* = *λ*_*n*_(*z*), *n* = 1,2,…,*N*_1_ + *N*_2_, are determined as the roots of the determinant:

1 $$\det (\lambda {\bf{I}}-{\boldsymbol{\Xi }}(z))=0$$

Here **I** is the unitary (*N*_1_ + *N*_2_) × (*N*_1_ + *N*_2_) matrix and matrix

2 $${\boldsymbol{\Xi }}(z)=\left(\begin{array}{cc}{{\boldsymbol{\Lambda }}}_{1}+{{\boldsymbol{\Delta }}}_{1}(z) & {{\boldsymbol{\Delta }}}_{12}(z)\\ {{\boldsymbol{\Delta }}}_{12}^{\dagger }(z) & {{\boldsymbol{\Lambda }}}_{2}+{{\boldsymbol{\Delta }}}_{2}(z)\end{array}\right)$$

includes submatrices determined by the original CWLs of Fibre 1 and Fibre 2, ${{\boldsymbol{\Lambda }}}_{j}=\{{\lambda }_{j{n}_{j}}+\frac{i}{2}{\gamma}_{jn_j}\}$, couplings inside each of the fibre caused by bending, ${{\boldsymbol{\Delta }}}_{j}(z)=\{{{\rm{\delta }}}_{{m}_{j}{n}_{j}}^{(j)}(z)\}$, and interfibre couplings ${{\boldsymbol{\Delta }}}_{12}(z)=\{{{\rm{\delta }}}_{{m}_{1}{n}_{2}}^{(12)}(z)\}$, ${m}_{j},{n}_{j}=1,2,\ldots {N}_{j}$.

As in SNAP^24^, dramatically small nanometre and sub-nanometre scale variations of CWLs *λ*_*n*_(*z*) along the compound fibre waveguide can localise WGMs and induce an optical microresonator having eigenwavelengths *λ*_*qn*_ with axial quantum numbers *q*. Due to the smooth and small CWL variation and proximity of the localised WGM wavelengths *λ*_*qn*_ to *λ*_*n*_(*z*), the corresponding eigenmode can be presented as *E*_*qn*_(*x,y,z*) = Ψ_*qn*_(*z*)Ω_*n*_(*x,y,z*) where the transverse WGM distribution Ω_*n*_(*x,y,z*) is calculated at the CWL *λ*_*n*_(*z*) and depends on *z* parametrically slow^32^, and function Ψ_*qn*_(*z*) determines the axial dependence of the microresonator eigenmode amplitude and satisfies the one-dimensional wave equation^24^

3 $$\frac{{d}^{2}{\varPsi }_{n}}{d{z}^{2}}+{\beta }_{n}^{2}(z,\lambda ){\varPsi }_{n}=0,\,{\beta }_{n}(z,\lambda )=\frac{{2}^{3/2}\pi {n}_{r}}{{\lambda }_{n}^{3/2}}\sqrt{{\lambda }_{n}(z)-\lambda }$$

where *n*_*r*_ is the refractive index of fibres.

The coupling parameters *κ*_*qn*_(*z*) between WGM *E*_*qn*_(*x,y,z*) and the input-output wave in the microfibre is determined by their overlap integral. Commonly, the microfibre diameter is much smaller than the characteristic axial variation length of *E*_*qn*_(*x,y,z*). For this reason, similar to the analogous approximation in the SNAP platform^24,33^, the coupling parameters *κ*_*qn*_(*z*) are proportional to the values of *E*_*qn*_(*x,y,z*) at the axial coordinate *z* of the input-output microfibre. Then, calculations based on the Mahaux-Weidenmüller theory^34–36^ presented in Supplementary Material allowed us to express the transmission power *P*(*λ,z*) through the input-output microfibre coupled to the considered fibre configuration (Fig. 1b) as

4 $$P(z,\lambda )={\left|\frac{1+\mathop{\sum }\limits_{n=1}^{{N}_{1}+{N}_{2}}{D}_{n}^{\ast }(z){G}_{n}(z,z,\lambda )}{1+\mathop{\sum }\limits_{n=1}^{{N}_{1}+{N}_{2}}{D}_{n}(z){G}_{n}(z,z,\lambda )}\right|}^{2}$$

Here *G*_*n*_(*z*_1_,*z*_2_,*λ*) is the Green’s function of Eq. (3). Equation (4) generalises the expression for the transmission power previously derived in Ref. ^24^. As shown below, functions *D*_*n*_(*z*) can be expressed through and have characteristic values similar to the coupling *D*-parameters, which were experimentally measured previously and typically have the real and imaginary parts ~0.01 µm^−1^ ^24,33^. Close to the resonance condition, *λ* = *λ*_*qn*_, for sufficiently small losses and coupling, and separated CWLs *λ*_*n*_(*z*), only one Green’s function with number *n* contributes to the sums in Eq. (4). Then, Eq. (4) coincides with that previously derived in Ref. ^24^. However, generally, the contribution of more than one term to the sums in Eq. (4) may be significant.

Before the detailed description of the spectrograms in Figs. 2b and 3b, we note that the transmission power plots in these figures characterise the CWLs of the coupled fibre system determined by Eq. (1) viewed by the input-output microfibre and, subsequently, OSA. Therefore, the CWLs of Fibre 2, which are the solutions of Eq. (1) but uncoupled from Fibre 1 cannot be seen by the OSA. On the other hand, the number of CWLs which can show up in the coupling region can be as many as *N*_1_ + *N*_2_, i.e. significantly greater than the number *N*_1_ of visible uncoupled CWLs of Fibre 1 (see Fig. 2b as an example).

To clarify the effect of coupling between WGMs in adjacent fibres, we consider the two-mode approximation, *N*_1_ = *N*_2_ = 1, assuming that the wavelength *λ* of the input light is close to an unperturbed single WGM CWL ${\lambda }_{11}+\frac{i}{2}\gamma$ of Fibre 1 and a single CWL ${\lambda }_{21}+\frac{i}{2}\gamma$ of Fibre 2 having the same imaginary part. Consequently, in Fig. 1b we now set *n*_1_ = *n*_2_ = 1. We neglect the effect of the CWL variation due to the fibre bending^27^, which is usually smaller than the effect of fibre coupling, setting ${\delta }_{11}^{(j)}=0$. Then, the CWLs *λ*_1_(*z*) and *λ*_2_(*z*) of the compound fibre are found from Eq. (1) as

5 $${\lambda }_{1,2}(z)=\frac{1}{2}({\lambda }_{11}+{\lambda }_{21})+i\gamma \pm \sqrt{\frac{1}{4}{({\lambda }_{11}-{\lambda }_{21})}^{2}+{({\delta }_{11}^{(12)}(z))}^{2}}$$

The dependence on the transverse coordinates *x* and *y* (Fig. 1b) of the compound WGM corresponding to CWLs *λ*_*j*_(*z*) can be calculated as follows. We introduce the unperturbed WGMs in Fibre 1 and 2 (considered unbent and uncoupled) calculated at their CWLs *λ*_11_ and *λ*_21_ as ${\varOmega }_{1}^{(1)}(x,y)$ and ${\varOmega }_{1}^{(2)}(x,y)$. Then, in the two-mode approximation, the compound modes generated by weak coupling of modes ${\varOmega }_{1}^{(1)}(x,y)$ and ${\varOmega }_{1}^{(2)}(x,y)$ are determined as^37^

6 $$\begin{array}{c}{\varOmega }_{1}(x,y,z)=\,\cos (\alpha ){\varOmega }_{1}^{(1)}(x,y)+\,\sin (\alpha ){\varOmega }_{1}^{(2)}(x,y)\\ {\varOmega }_{2}(x,y,z)=-\,\sin (\alpha ){\varOmega }_{1}^{(1)}(x,y)+\,\cos (\alpha ){\varOmega }_{1}^{(2)}(x,y)\\ {\rm{tan}}(2\alpha )=\frac{2{\delta }_{11}^{(12)}(z)}{{\lambda }_{11}-{\lambda }_{21}}\end{array}$$

Consequently, the coupling parameters to the microfibre entering Eq. (4) at coordinate *z* are

7 $${D}_{1,2}(z)=\frac{D}{2}\left(1\pm \frac{{\lambda }_{1}-{\lambda }_{2}}{\sqrt{{({\lambda }_{1}-{\lambda }_{2})}^{2}+4{({\delta }_{11}^{(12)}(z))}^{2}}}\right)$$

where *D* is the *z*-independent coupling parameter between the input-output microfibre and Fibre 1 (refs. ^24,33^).

To map the bent fibre axial profile *h*(*z*) to the CWL envelope profiles of the induced microresonators, we have to determine the relation between *h*(*z*) and coupling coefficient ${\delta }_{11}^{(12)}(z)$. Similar to calculations in Refs. ^38,39^, for the smooth and small *h*(*z*) considered here, we find

8 $${\delta }_{11}^{(12)}(z)={\delta }_{0}\exp\left (-\frac{2\pi }{\lambda }{({n}_{r}^{2}-1)}^{1/2}h(z)\right)$$

where *δ*_0_ is *z*-independent. Assuming the simplest profile of the bent fibre having the curvature radius *R* as

9 $$h(z)={z}^{2}/2R$$

for silica fibres with *n*_*r*_ = 1.44, we estimate the FWHM of ${\delta }_{11}^{(12)}(z)$ as ${z}_{FWHM} \sim 0.5{(\lambda R)}^{1/2}$. At *λ* ~ 1.55 µm and *R* ~ 30 mm of our experiment, we have *z*_*FWHM*_ ~ 100 µm. From Eqs. (5) and (8), we find that the FWHM of the CWL, depending on the value of *λ*_11_ − *λ*_12_, is between *z*_*FWHM*_ and 2*z*_*FWHM*_ which is only in qualitative agreement with the microresonator FWHM *z*_*FWHM*_ ~ 250 µm found from experimental data in Fig. 3c and d.

The results of our numerical modelling in the two-mode approximation considered based on Eqs. (3)–(9) are shown in Fig. 3e and f. To fit the experimental data, we set the average CWL 0.5(*λ*_11_ + *λ*_12_) = 1.55 µm, the CWL difference *λ*_11_ − *λ*_12_ = 0.05 nm in Fig. 3e and *λ*_11_ − *λ*_12_ = −0.05 nm in Fig. 3f, coupling parameter *D* = −0.01 + 0.01*i* µm^−1^ (refs. ^24,33^), Q-factor *Q* = 10^6^, the microresonator FWHM *z*_*FWHM*_ ~ 250 µm and its spectral height ~0.15 nm, similar to these values found from Fig. 3c, d.

The experimental spectrograms in Fig. 3c, d and theoretical spectrograms in Fig. 3e and f look nicely similar. However, important differences between them should be noted. From Eqs. (8) and (9), the FWHM value *z*_*FWHM*_ ~ 250 µm corresponds to the Fibre 2 curvature radius *R* ~ 66 mm, which is twice as large as that measured from the fibre image shown in Fig. 3a. We suggest that the difference is caused by the deviation of the shape of Fibre 2 from parabolic in the coupling region as well as by the fibre misalignment. The additional deformation of fibres may be induced by their electrostatic attraction and pressuring, which are not visible in Fig. 3a. Our suggestion is confirmed by the experimental profiles of the induced microresonator envelopes and CWL shapes in Fig. 3c, d which, as compared to those in the theoretical spectrograms in Fig. 3e, f, have larger side slopes and are flatter in the middle. Next, we notice that, in the theoretical spectrograms, the CWL wavelength profiles are more mirror-symmetric to the microresonator envelopes with respect to the horizontal line (following Eq. (5)), while, in the experimental spectrograms, the lower CWL profiles are shallower than the microresonator envelopes. We suggest that this deviation can be eliminated by taking into account the coupling with other WGMs ignored in the two-mode approximation considered.

### Tunability

**Fig. 4:**
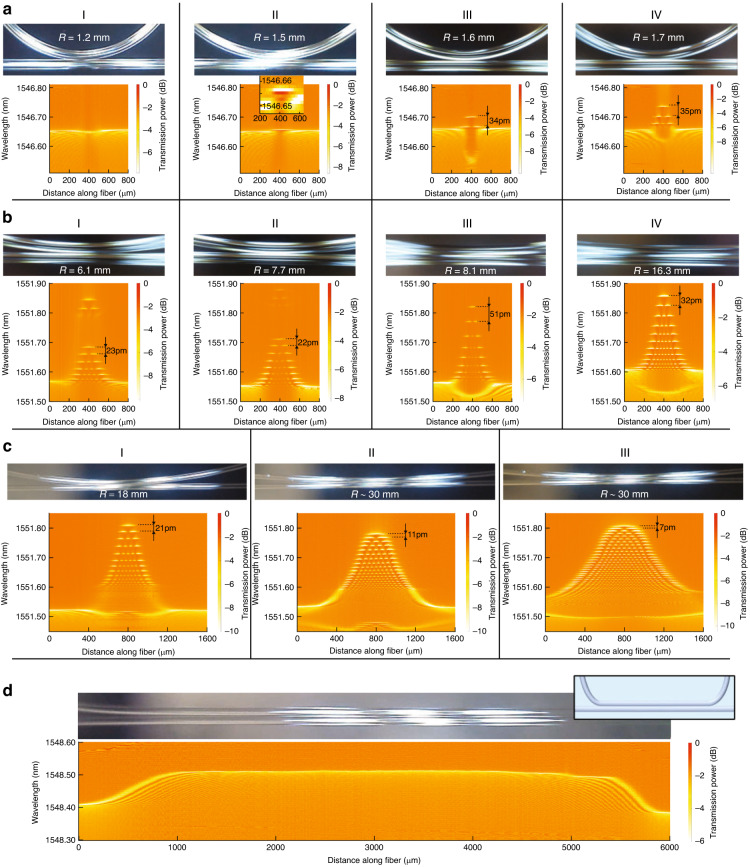
Tunability of microresonators. **a** Spectrograms of induced microresonators for small curvature radius of Fibre 2–1 mm. **b** and **c** Spectrograms of induced microresonators for a lager radius of Fibre 2–10 mm. **d** Spectrogram of a 5 mm long microresonator induced by touching straight Fibre 1 and Fibre 2 which was preliminary permanently bent at the ends as shown in the inset

Bending and translating the tails of Fibre 1 and Fibre 2 side-coupled to each other as illustrated in Fig. 1 allowed us to tune the dimensions of the fibre coupling region and thereby tune the dimensions of created microresonators. As in the previous experiments, we used 125 µm optical fibres. We investigated the cases of the smallest microresonators containing a few wavelength eigenvalues and having the characteristic axial dimensions of hundred microns (Fig. 4a), as well as larger microresonators with dimensions of several hundred microns (Fig. 4b, c) and the largest microresonator having the axial length of 5 millimetres (Fig. 4d). For detail visibility, the spectrograms shown in Fig. 4 are magnified in Section 3 of the Supplementary Information, which also includes the exemplary plots of transmission spectra extracted from these spectrograms at different positions along the fibre axis. In the figures of the same section, we present the insets showing the transmission power resonances at the axial coordinates close to the nodes of WGM eigenstates. The characteristic widths of these resonances suggest that the Q-factor of created microresonators does not depend on bending radius *R* and its value is comparable with or larger than 10^6^.

Considering the smallest microresonators, we monitored the process of their creation. Side-coupling of a straight Fibre 1 and Fibre 2 bent with a sufficiently small curvature radius of ~1 mm introduced small perturbation in CWLs shown in Fig. 4a, I. Increasing the fibre radius further, we arrived at the microresonator with a single eigenwavelength (Fig. 4a, II)). The inset inside the spectrogram of Fig. 4a, II, which magnifies the region near this eigenwavelength, shows that the axial dimension of the corresponding eigenmode is ~200 µm. Remarkably, except for the axial dimension of localised WGMs with uniform magnitude in specially designed bat microresonators^39,40^, this dimension (which expansion is critical, e.g. for QED applications^41^) is the record large FWHM of the WGM amplitude antinode area demonstrated in microresonators to date. The Q-factor of this microresonator (limited by the 1.3 pm resolution of the OSA used) estimated in Section 3 of the Supplementary Information was >10^6^. Comparison of the spacing ∆*λ*_01_ between the eigenwavelength of the fundamental and second axial modes of microresonators shown in Fig. 4a(II) (∆*λ*_01_ = 34 pm) and in Fig. 4a(III) (∆*λ*_01_ = 35 pm) shows that, while the microresonator CWL height increases with bent fibre radius *R*, the value of ∆*λ*_01_, which characterised the local CWL behaviour near the top of the microresonator, remains practically unchanged.

Larger bending radii of Fibre 2 having the order of 10 mm led to the creation of microresonators with millimetre-order axial dimensions having the spectrograms shown in Fig. 4b, c. In these cases, as in Fig. 4(a), the microresonator CWL height grows with *R*. However, in Fig. 4(b), the value of eigenwavelength spacing ∆*λ*_01_ first slightly decreases with growing *R* (compare Fig. 4b(I) and 4b(II)), then grows together with *R* (Fig. 4b(III), and then again decreases with increasing *R* in Fig. 4b(IV). In particular the value of ∆*λ*_01_ achieves 51 pm in Fig. 4b(III). We suggest that the observed nonmonotonic dependence of ∆*λ*_01_ on *R* is caused by the reasons noted in the previous section, i.e. by the local fibre deformation caused by pressuring and by electrostatic attraction of fibres. The deviation of the CWL variation from the parabolic in the demonstrated microresonators is reflected by the nonuniformity of their FSR. In several cases (e.g. in Fig. 4c) the FSR is close to constant, which suggests that these microresonators can be used, e.g. as tuneable optical frequency comb generators^42^. We note that the behaviour of the CWLs and microresonators envelopes in most of these spectrograms cannot be accurately described by the two-mode approximation considered above. Of particular interest is the spectrogram shown in Fig. 4c(II). At first sight, the envelop of the microresonator in this spectrogram is the continuation of the CWL of Fibre 1 (compare with Fig. 3c, d). Unexpectedly, the axial WGM localisation in this microresonator (caused by the WGM reflection from the CWL-generated turning points^24^) sharply dissolves inside the microresonator area. The theoretical explanation of this effect is beyond the scope of this paper.

To create longer microresonators, we, first, permanently bent the tails of Fibre 2 as illustrated in the inset of Fig. 4d. This allowed us to arrive at an arbitrarily large curvature radius of this fibre including its straight shape between the bent tails. As an example, Fig. 4d shows the spectrogram of a 5 mm long microresonator. Though the eigenwavelength width of this microresonator is greater than its free spectral range, i.e. it belongs to the class of white light WGM resonators^43^, we suggest that, in contrast to the lossy microresonators induced by side-coupled cleaved straight fibres (Fig. 2), its Q-factor is similar to that of the smaller microresonators with spectrograms shown in Figs. 3 and 4.

## Discussion

The high Q-factor WGM tuneable optical microresonators induced in side-coupled optical fibres discovered in this paper enable a range of exciting generalisations and applications. Further extension of tuning flexibility can be achieved by applying different boundary conditions at the fibre tails (Fig. 1a), different interfibre touching stresses, and different preliminary permanent fibre bending. An interesting topic of the future research consists in design and realisation of advanced systems modifying our original proof-of-concept setup to arrive at its better flexibility enabling accurately reproducible induction of microresonators with predetermined spectra.

Configurations of fibres, which are potentially attractive for future research and applications can be realised from silica fibres considered in this paper as well as fibres fabricated of other materials, including microcapillary fibres and fibres exhibiting high nonlinearity. Side coupling of bent fibres and WGM microresonators can be used for microresonator tuning. Multiple side coupled straight, bent, and twisted optical fibres can present a new type of photonic molecules^44^.

While the model of two coupled CWLs developed here qualitatively explains some characteristic features of the experimentally measured spectrograms, the complete explanation and quantitative fitting of the experimental data should include the effect of several coupled CWLs and be based on the further development of the coupled wave theory. In particular, it is interesting to develop the detailed electromagnetic theory of the coupled fibre configurations following the corresponding theories for spherical microresonators including the detailed description of coupling and surface scattering losses^38,45,46^. The future theory should also allow us to express the fibre profiles and deformation in the region of coupling through the values of forces and moments applied to the fibre tails (Fig. 1a) including the effect of electrostatic fibre attraction.

We suggest that the Q-factor of demonstrated microresonators (measured here as ~10^6^) can be further increased to the values exceeding 10^8^ if a fixed submicron-wide gaps between coupled fibres and input-output microfibre (rather than their direct contact considered here) is introduced^8^. While such large Q-factors are not required for the realisation of a range of optical tuneable resonant microdevices including delay lines^30^, signal processors^25^, and microlasers^19–21^, they may be important for the realisation of frequency comb generators with tuneable repetition rate^15,16,42^, as well as for the cavity QED^8,11,12^ and optomechanical applications^13,14^.

## Materials and methods

### Experimental

The fibres used in our experiments were the commercial 125 µm diameter single-mode silica fibres. The fibres were mechanically uncoated and cleaned in isopropanol. The measurements of spectra of side-coupling optical fibres illustrated in Fig. 1 were performed with the Luna-5000 Optical Vector Analyzer (Luna OVA) having the resolution 1.3 pm at telecommunication wavelengths ~1.55 µm. For this purpose, we fabricated a biconical fibre taper having a micron waist diameter whose ends were connected to the output and input of the Luna OVA. The taper was drawn in the NTT ceramic heater CMH-7019 by direct fibre pulling. The waist of the taper (microfibre) was oriented normal to the axes of fibres and put in direct contact with one of the coupled fibres. The Luna OVA measured the Jones matrix of light transmitted through the considered system as a function of wavelength while the microfibre was translated along the coupled fibres and periodically touched one of the fibres with a 2 µm period. In our measurements, we collected the spectra as a function of wavelength *λ* measured along the 10 nm bandwidth near 1.55 µm radiation wavelength. The spectral data were collected along the mm-order axial length, which included the region where fibres couple to each other. After that, the Jones matrices were diagonalised using the approach described in ref. ^47^. As a result, the spectrograms of two separated polarisations of light were obtained. Since the separation was performed analytically rather than using polarisation controllers, we did not distinguish the TM and TE polarisations. An example of measured spectrograms for different polarisations is given in the Supplementary Information.

### Theoretical

The detailed derivation of the expression for the transmission power *P*(*z,λ*) (Eq. (4)) using the Mahaux-Weidenmüller theory^34–36^ is presented in the Supplemental Information. Calculation of the Green’s function of Eq. (3) entering Eq. (4) and P(*z,λ*) was performed numerically using a Mathcad code.

### Supplementary information

Supplementary information file

## Data Availability

Data underlying the results presented in this paper may be obtained from the authors upon reasonable request.

## References

[1] Bogaerts W (2020). Programmable photonic circuits. Nature.

[2] Siew SY (2021). Review of silicon photonics technology and platform development. J. Lightwave Technol..

[3] Lu ZQ (2017). Performance prediction for silicon photonics integrated circuits with layout-dependent correlated manufacturing variability. Opt. Express.

[4] Lian CY (2022). Photonic (computational) memories: tunable nanophotonics for data storage and computing. Nanophotonics.

[5] Ko JH (2022). A review of tunable photonics: optically active materials and applications from visible to terahertz. iScience.

[6] Savchenkov AA (2003). Tunable filter based on whispering gallery modes. Electron. Lett..

[7] Armani D (2004). Electrical thermo-optic tuning of ultrahigh-*Q* microtoroid resonators. Appl. Phys. Lett..

[8] Pöllinger M (2009). Ultra-high-*Q* tunable whispering-gallery-mode microresonator. Phys. Rev. Lett..

[9] Sumetsky M, Dulashko Y, Windeler RS (2010). Super free spectral range tunable optical microbubble resonator. Opt. Lett..

[10] Kovach A (2020). Optically tunable microresonator using an azobenzene monolayer. AIP Adv..

[11] Buck JR, Kimble HJ (2003). Optimal sizes of dielectric microspheres for cavity QED with strong coupling. Phys. Rev. A.

[12] Pfeifer H (2022). Achievements and perspectives of optical fiber Fabry–Perot cavities. Appl. Phys. B.

[13] Bahl G (2011). Stimulated optomechanical excitation of surface acoustic waves in a microdevice. Nat. Commun..

[14] Lambert NJ (2020). Coherent conversion between microwave and optical photons—an overview of physical implementations. Adv. Quant. Technol..

[15] Bao HL (2019). Laser cavity-soliton microcombs. Nat. Photon..

[16] Chang L, Liu ST, Bowers JE (2022). Integrated optical frequency comb technologies. Nat. Photon..

[17] Wang XY (2017). Continuously tunable ultra-thin silicon waveguide optical delay line. Optica.

[18] Zhang W, Yao JN, Zhao YS (2016). Organic micro/nanoscale lasers. Acc. Chem. Res..

[19] Zhu S (2018). All-optical Tunable microlaser based on an ultrahigh-*Q* erbium-doped hybrid microbottle cavity. ACS Photon..

[20] Zhu S (2019). Tunable Brillouin and Raman microlasers using hybrid microbottle resonators. Nanophotonics.

[21] Yang X (2022). Fiber optofluidic microlasers: structures, characteristics, and applications. Laser Photon. Rev..

[22] Greuter L (2014). A small mode volume tunable microcavity: development and characterization. Appl. Phys. Lett..

[23] Flågan S (2022). Microcavity platform for widely tunable optical double resonance. Optica.

[24] Sumetsky M (2012). Theory of SNAP devices: basic equations and comparison with the experiment. Opt. Express.

[25] Sumetsky M (2019). Optical bottle microresonators. Prog. Quant. Electron..

[26] Dmitriev, A., Toropov, N. & Sumetsky, M. Transient reconfigurable subangstrom-precise photonic circuits at the optical fiber surface. 2015 IEEE Photonics Conference (IPC). 1–2 (IEEE, Reston, VA, USA, 2015).

[27] Bochek D (2019). SNAP microresonators introduced by strong bending of optical fibers. Opt. Lett..

[28] Sumetsky M, Dulashko Y (2010). Radius variation of optical fibers with angstrom accuracy. Opt. Lett..

[29] Kudashkin DV (2020). Reflection of whispering gallery modes propagating on a surface of an optical fiber from its cleave. Opt. Express.

[30] Sumetsky M (2013). Delay of light in an optical bottle resonator with nanoscale radius variation: dispersionless, broadband, and low loss. Phys. Rev. Lett..

[31] Toropov N (2021). Microresonator devices lithographically introduced at the optical fiber surface. Opt. Lett..

[32] Snyder, A. W. & Love, J. D. Optical Waveguide Theory. (Springer, New York, 1983).

[33] Vitullo DLP (2020). Coupling between waveguides and microresonators: the local approach. Opt. Express.

[34] Mahaux, C. & Weidenmüller, H. A. Shell-Model Approach to Nuclear Reactions. (Amsterdam, London, North-Holland Pub. Co., 1969).

[35] Dittes FM (2000). The decay of quantum systems with a small number of open channels. Phys. Rep..

[36] Sumetsky M (2019). Mahaux-Weidenmüller approach to cavity quantum electrodynamics and complete resonant down-conversion of the single-photon frequency. Phys. Rev. A.

[37] Cohen-Tannoudji, C., Diu, B. & Laloë, F. Quantum Mechanics. (John Wiley & Sons, New York, 1977).

[38] Little BE, Laine JP, Haus HA (1999). Analytic theory of coupling from tapered fibers and half-blocks into microsphere resonators. J. Lightwave Technol..

[39] Sumetsky M (2021). Fundamental limit of microresonator field uniformity and slow light enabled ultraprecise displacement metrology. Opt. Lett..

[40] Yang, Y., Crespo-Ballesteros, M. & Sumetsky, M. Experimental demonstration of a bat microresonator. 2021 Conference on Lasers and Electro-Optics Europe & European Quantum Electronics Conference (CLEO/Europe-EQEC). (IEEE, Munich, Germany, 2021).

[41] Chang DE (2018). *Colloquium*: quantum matter built from nanoscopic lattices of atoms and photons. Rev. Mod. Phys..

[42] Suchkov SV, Sumetsky M, Sukhorukov AA (2017). Frequency comb generation in SNAP bottle resonators. Opt. Lett..

[43] Savchenkov AA, Matsko AB, Maleki L (2006). White-light whispering gallery mode resonators. Opt. Lett..

[44] Li YC (2017). Whispering gallery mode hybridization in photonic molecules. Laser Photon. Rev..

[45] Matsko AB, Ilchenko VS (2006). Optical resonators with whispering-gallery modes-part I: basics. IEEE J. Sel. Top. Quant. Electron..

[46] Gorodetsky ML, Pryamikov AD, Ilchenko VS (2000). Rayleigh scattering in high-*Q* microspheres. J. Opt. Soc. Am. B.

[47] Crespo-Ballesteros M (2019). Four-port SNAP microresonator device. Opt. Lett..

