# Supplementary material for: High Q-factor reconfigurable microresonators induced in side-coupled optical fibres
Source: Light Sci Appl. 2023 Aug 18;12:197. doi: 10.1038/s41377-023-01247-7 (PMC10439148; doi:10.1038/s41377-023-01247-7)
Supplement: Supplementary file 1 — Supplementary information file [file 41377_2023_1247_MOESM1_ESM.docx]

**Supplemental Information for**

**High Q-factor reconfigurable microresonators induced in side-coupled optical fibres**

Victor Vassiliev and Michael Sumetsky*

Aston Institute of Photonic Technologies, Aston University, Birmingham B4 7ET, UK

*Corresponding author, email: [m.sumetsky@aston.ac.uk](mailto:m.sumetsky@aston.ac.uk)

S1. Expression for the transmission power

We calculate the resonant transmission power through the microfiber coupled to the structure of side-coupled optical fibres illustrated in Fig. S1a and S1b in two steps. First, we introduce the discrete eigenwavelengths of the microresonator in the compound fibre system, $\lambda_{m}+{\frac{i}{2}\gamma}_{m}$, $m=1,2,\ldots,M$and coupling coefficients $\kappa_{m}(z)$ between the corresponding eigenmodes and the input-output waves in the microfiber with wavelength $\lambda$ positioned at axial coordinate $z$ (Fig. S1c). In this formulation, our system is a particular case of systems considered previously in quantum mechanics ^1,2^ and photonics ^3^ where the analytical expression for the transmission power through such systems in the resonance approximation of our interest has been found. In the second step of our calculations, we renumerate the eigenwavelengths of the induced microresonator according to their belonging to CWLs quantum wells $\lambda_{n}\left( z \right)$ forming localised WGMs (Fig. S1d). This allows us to express the transmission power through the Green’s functions $G_{n}(z_{1},z_{2},\lambda)$, of Eq. (3) of the main text, which determines the eigenmodes of the induced microresonator formed by the CWL $\lambda_{n}\left( z \right)$. The details of our calculations are as follows.

First, we calculate the transmission power $P(\lambda,z)$ of our system by applying the Mahaux-Weidenmüller formula ^1-3^:

, (S1)

where

 (S2)


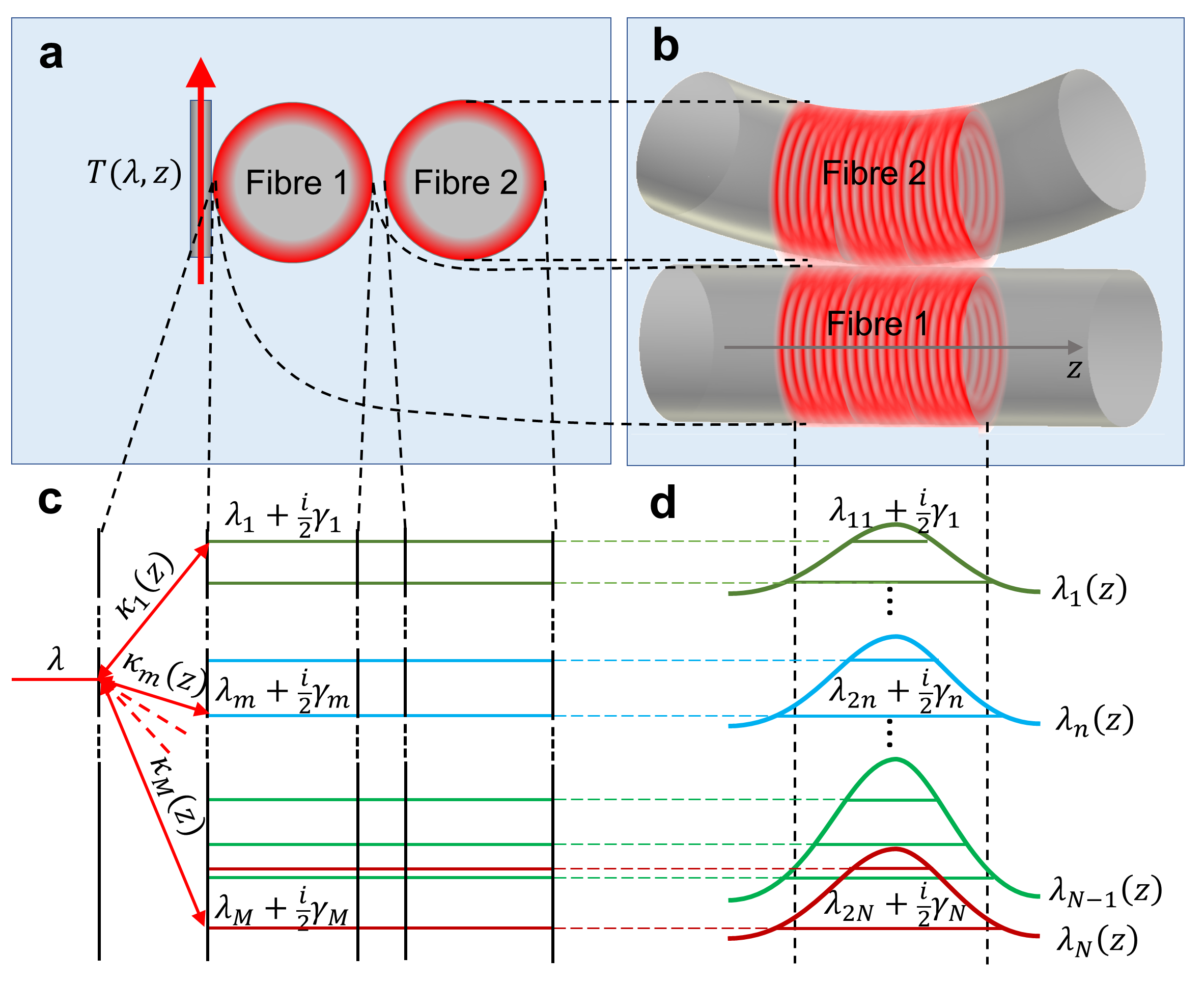


**Fig. S1**. **a** A cross-section of the side-coupled fibre system at the point of contact with the input-output microfibre. **b** The profile of the same fibre system. **c** The eigenwavelengths of the compound fibre system $\lambda_{m}+{\frac{i}{2}\gamma}_{m}$ numerated from $m=1$ to $m=M$. **d** The same eigenwavelengths renumerated according to their belonging to CWLs quantum wells $\lambda_{n}\left( z \right)$ forming localised WGMs.

It is assumed in Eq. (S1) that the coupling to the input-output waveguide does not introduce the shifts of the eigenwavelengths ^2^ which will be added later. We simplify the expression for the transmission power by expanding the inverse matrix in Eq. (S1) as follows:

 (S3)

Substituting this expression into Eq. (S1), we find:

 (S4)

We separate the series of eigenwavelengths $\lambda_{m}+{\frac{i}{2}\gamma}_{m}$ and coupling coefficients $\kappa_{m}(z)$ by their correspondence to CWLs $\lambda_{n}\left( z \right)$ entering Eq. (3) of the main text. For this purpose, we rewrite these parameters as $\lambda_{qn}+{\frac{i}{2}\gamma}_{n}$ and $\kappa_{qn}(z)$, where $q$ is the axial quantum number of the eigenmode $E_{qn}\left( x,y,z \right)=\Psi_{qn}\left( z \right)\Omega_{n}\left( x,y,z \right).$ Here $\Psi_{qn}(z)$ satisfies Eq. (3) and $\Omega_{n}\left( x,y,z \right)$ is a parametrically slow function of the axial coordinate $z$. Substituting $\gamma_{m}\to\gamma_{n}$ we assume that the material losses do not depend on the axial quantum number $q$. Then, similar to the arguments of Ref. ^4^ (see Eq. (13) in this reference), the coupling coefficients can be factorised as $\left| \kappa_{qn}(z) \right|^{2}={2iD}_{n}(z)\left| \Psi_{qn}(z) \right|^{2}$. Using the expression for the Green’s function of Eq. (3),

 , (S5)

we rewrite Eq. (S4) as

$P(\lambda,z)=\left|\frac{1+\sum_{n=1}^{N} D_{n}^{*}(z)G_{n}(z,z,\lambda)}{1+\sum_{n=1}^{N} D_{n}(z)G_{n}(z,z,\lambda)}\right|^{2}$. (S6)

To identify the physical meaning of parameters $D_{n}(z)$, we recall the expression for the transmission power of a SNAP microresonator under the assumption of a single CWL contribution ($N=1$) and lossless coupling to the input-output microfiber ^24^:

$P_{1}(\lambda,z)=\left|\frac{1+D_{1}^{*}G_{1}(z,z,\lambda)}{1+D_{1}G_{1}(z,z,\lambda)}\right|^{2}$ (S7)

Here complex parameter $D_{1},$ which was experimentally measured and analysed previously ^4, 5^, determines the coupling to the input-output microfiber as well as the WGM phase shift due to this coupling. Importantly, while the imaginary part of $D_{n}(z)$ contributes to the widths of the resonances, its real part (not taken into account in the original Eq. (S1)) determines the WGM phase shifts caused by the coupling to the input-output microfiber.

S2. Numerical separation of polarisations

In our experiments, the spectra of side-coupled fibres were obtained with Luna-5000 Optical Vector Analyser (OVA), which measured the Jones matrices of the transmitted light. We used the Jones matrices to determine the polarisation states of the side-coupled optical fibres using the numerical approach described in Ref ^6^. In this approach, the Jones matrix of our device under test (DUT), $J_{DUT}(\lambda)$, is expressed, within an inessential constant factor, through the Jones matrix measured by OVA, $J_{OVA}(\lambda)$, by the relation

$J_{DUT}(\lambda)=U_{out}J_{OVA}(\lambda)U_{in}$ (S8)

Here $U_{in}$ and $U_{out}$ are unitary matrices which account for the field transformation in the input and output of the OVA and optical fibres, as well as for the unitary transformation of the Jones matrix of our device $J_{DUT}(\lambda)$ to the diagonal form so that the diagonal elements of this matrix determines its polarisation states. In the spectral bandwidth of our concern, matrices $U_{in}$ and $U_{out}$ can be assumed wavelength independent. These matrices contain seven free parameters which are determined by minimisation of the off-diagonal elements of $J_{DUT}(\lambda)$. The remaining diagonal elements of $J_{DUT}(\lambda)$ determined the transmission amplitudes for each polarisation state. As an example, Fig. S2 compares the spectrograms of the side-coupled optical fibres found for different polarisations within the same spectral bandwidth. Spectrogram a in Fig. S2 replicates the spectrogram shown in Fig. 3b of the main text. For comparison, spectrogram b is calculated for the orthogonal polarisation of light. It is seen that the CWLs in these spectrograms are located at different wavelengths and exhibit noticeably different coupling behaviour.


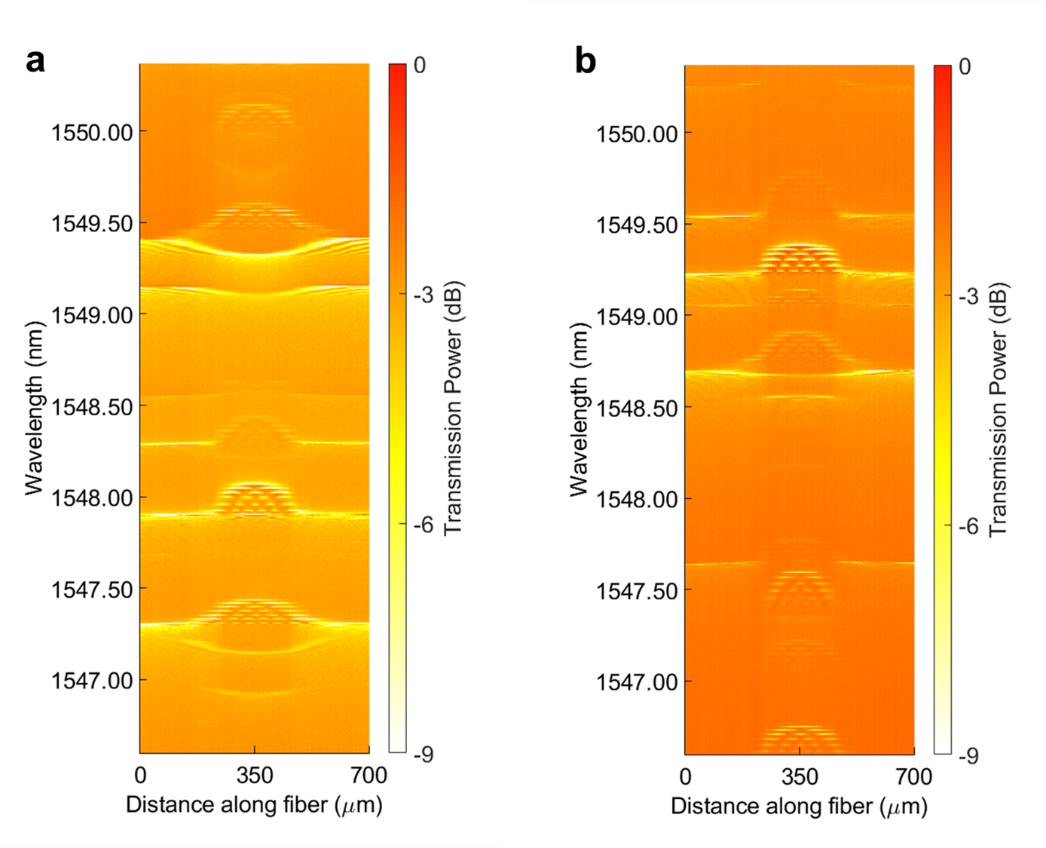


**Fig. S2**. **a** and **b** Spectrograms for two orthogonal polarisation states of the side coupled optical fibres considered in the main text of the paper.

S3. Magnified spectrograms of Fig. 4 with exemplary plots of transmission power spectra

In Figs. S3-S6 we present the magnified spectrograms shown in Fig. 4 of the main text and exemplary transmission power spectral cuts extracted from these spectrograms. Figs. S3, S4, S5, and S6 include magnified spectrograms from Figs. 4a, b, c, and d, respectively. In all figures, we chose three spectral cuts of the transmission power spectra at equal distance from each other where the last cut coincided with the microresonator centre. In Fig. 4a and b these cuts correspond to the axial coordinates *z* = 200, 300, and 400 µm. In Fig. 4c these cuts correspond to the axial coordinates *z* = 600, 700, and 800 µm. In Fig. 4d these cuts correspond to the axial coordinates *z* = 1000, 2000, and 3000 µm. To estimate the Q-factor from each spectrogram, we chose the narrowest dip in the presented spectral cuts with the smallest FWHM. The transmission power spectra near these resonance dips are magnified in the insets of some of these cut spectra. The spectral cut with the smallest resonance dip was found separately for the inset of the spectrogram a(II) in Fig. 4, which is magnified in Fig. S3. Due to the restrictions set by the resolution of the optical spectrum analyser used (Luna OVA-5000) of 1.3 pm at wavelengths ~ 1.55 µm considered, the maximum Q-factor we were able to measure was ~ 1.55 µm/1.3 pm, i.e., slightly greater than 10^6^. The plots of resonances in the insets shown in Figs. S3-S5 suggest that the Q-factors of all created microresonators did not noticeably change with tuning and are comparable with or greater than 10^6^.





**Fig. S3.** Magnified spectrograms of Fig. 4a with transmission power spectral cuts at the axial coordinates *z* = 200, 300, and 400 µm. The spectral cut used to find the narrowest resonance dip in the inset in Fig. II was chosen at *z* = 334 µm.


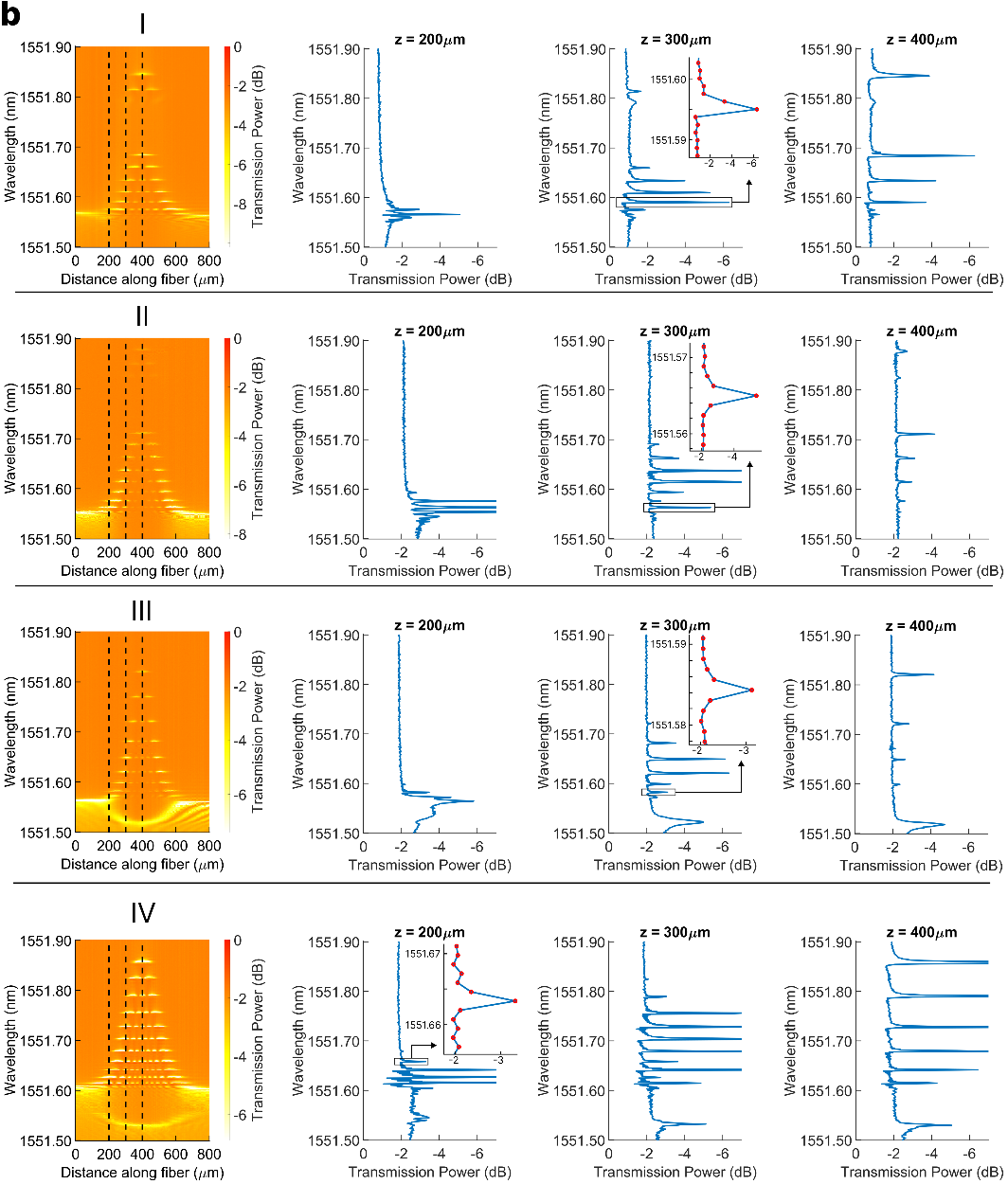


**Fig. S4.** Magnified spectrograms of Fig. 4b with transmission power spectral cuts at the axial coordinates *z* = 200, 300, and 400 µm.


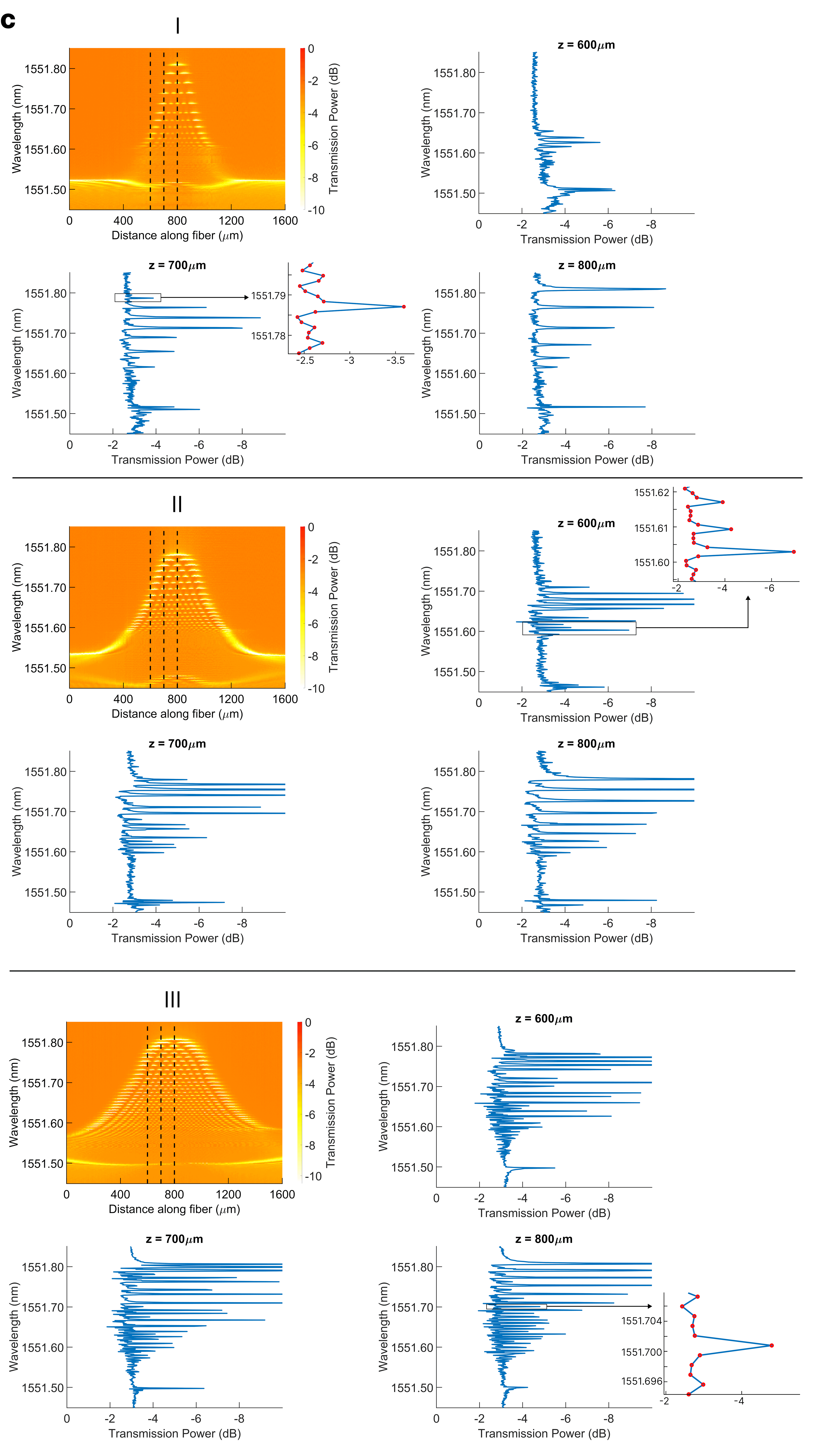


**Fig. S5.** Magnified spectrograms of Fig. 4c with transmission power spectral cuts at the axial coordinates *z* = 600, 700, and 800 µm.


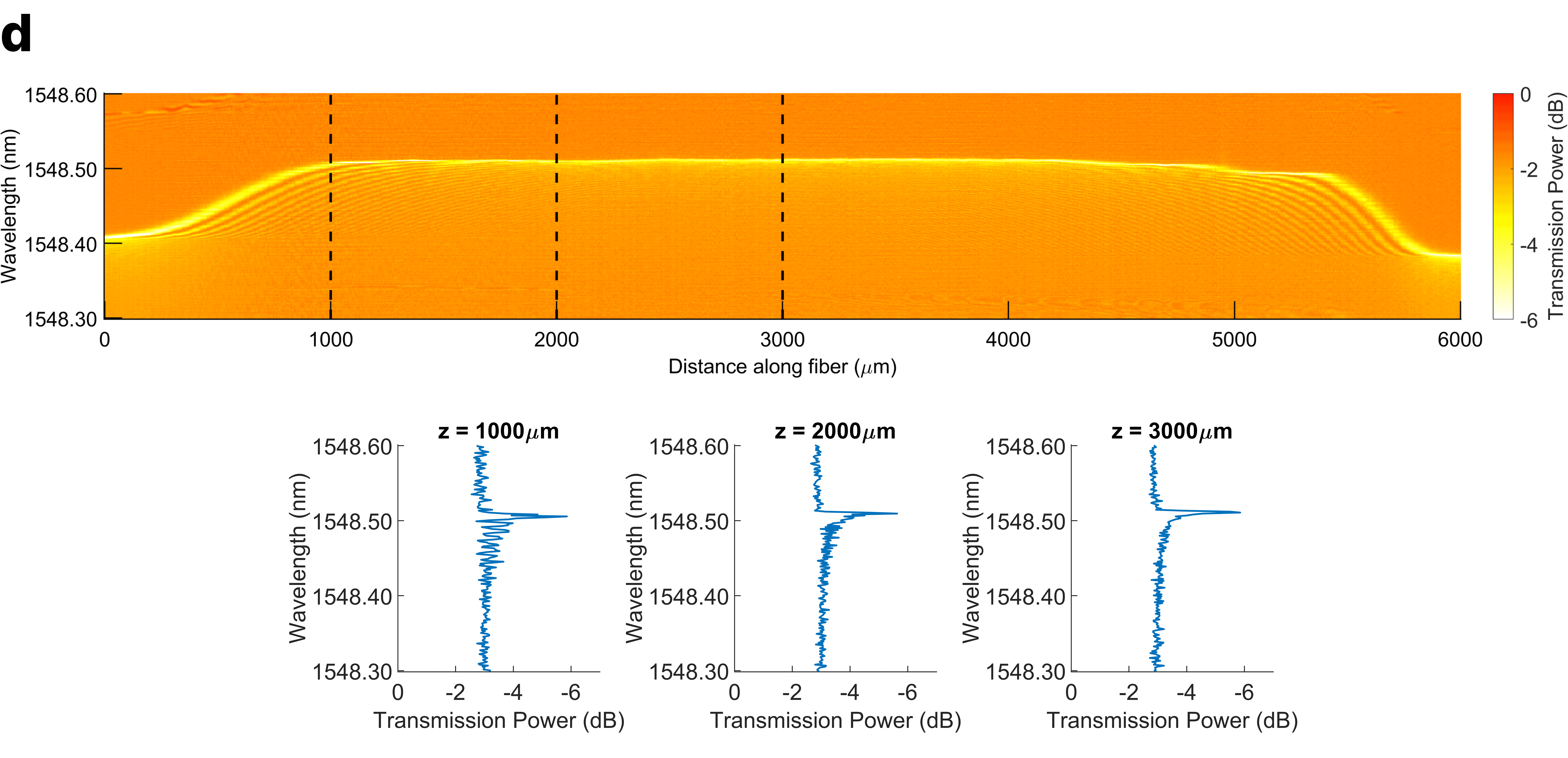


**Fig. S6.** The spectrogram of Fig. 4d with transmission power spectral cuts at the axial coordinates *z* = 1000, 2000, and 3000 µm.

References

1. Mahaux C. & Weidenmüller, H. A. *Shell-Model Approach to Nuclear Reactions* (North-Holland, Amsterdam, 1969).
2. Dittes, F.-M. The decay of quantum systems with a small number of open channels. *Phys. Rep.* **339**, 215-316 (2000).
3. Sumetsky, M. Mahaux-Weidenmüller approach to cavity quantum electrodynamics and complete resonant down-conversion of the single-photon frequency. *Phys. Rev. A* **100**, 013801 (2019).
4. Sumetsky, M. Theory of SNAP devices: basic equations and comparison with the experiment. *Opt. Express* **20**, 22537- 22554 (2012).
5. Vitullo, D. L. P., Zaki, S., Jones, D. E., Sumetsky, M. & Brodsky, M. Coupling between waveguides and microresonators: the local approach. *Opt. Express* **28**, 25908-25914 (2020).
6. Crespo-Ballesteros, M., Yang, Y., Toropov, N. & Sumetsky, M. Four-port SNAP microresonator device. *Opt. Lett*. **44**, 3498-3501 (2019).
